# Directional and general impairments in initiating motor responses after stroke

**DOI:** 10.1093/braincomms/fcad066

**Published:** 2023-03-20

**Authors:** Kayne Park, Matthew J Chilvers, Trevor A Low, Sean P Dukelow, Stephen H Scott

**Affiliations:** Centre for Neuroscience Studies, Queen’s University, Kingston, ON K7L 3N6, Canada; Department of Clinical Neurosciences, Hotchkiss Brain Institute, University of Calgary, Calgary, AB T2N 4N1, Canada; Department of Clinical Neurosciences, Hotchkiss Brain Institute, University of Calgary, Calgary, AB T2N 4N1, Canada; Department of Clinical Neurosciences, Hotchkiss Brain Institute, University of Calgary, Calgary, AB T2N 4N1, Canada; Centre for Neuroscience Studies, Queen’s University, Kingston, ON K7L 3N6, Canada; Department of Biomedical and Molecular Sciences, Queen’s University, Kingston, ON K7L 3N6, Canada

**Keywords:** visuospatial neglect, reaction time, Behavioral Inattention Test, reaching, imaging

## Abstract

Visuospatial neglect is a disorder characterized by an impairment of attention, most commonly to the left side of space in individuals with stroke or injury to the right hemisphere. Clinical diagnosis is largely based on performance on pen and paper examinations that are unable to accurately measure the speed of processing environmental stimuli—important for interacting in our dynamic world. Numerous studies of impairment after visuospatial neglect demonstrate delayed reaction times when reaching to the left. However, little is known of the visuospatial impairment in other spatial directions and, further, the influence of the arm being assessed. In this study, we quantify the ability of a large cohort of 204 healthy control participants (females = 102) and 265 individuals with stroke (right hemisphere damage = 162, left hemisphere damage = 103; mean age 62) to generate goal-directed reaches. Participants used both their contralesional and ipsilesional arms to perform a centre-out visually guided reaching task in the horizontal plane. We found that the range of visuospatial impairment can vary dramatically across individuals with some individuals displaying reaction time impairments restricted to a relatively small portion of the workspace, whereas others displayed reaction time impairments in all spatial directions. Reaction time impairments were observed in individuals with right or left hemisphere lesions (48% and 30%, respectively). Directional impairments commonly rotated clockwise when reaching with the left versus the right arms. Impairment in all spatial directions was more prevalent in right than left hemisphere lesions (32% and 12%, respectively). Behavioral Inattention Test scores significantly correlated (*r* = −0.49, *P* < 0.005) with reaction time impairments but a large portion of individuals not identified as having visuospatial neglect on the Behavioral Inattention Test still displayed reaction time impairments (35%). MRI and CT scans identified distinct white matter and cortical regions of damage for individuals with directional (insula, inferior frontal–occipital fasciculus and inferior longitudinal fasciculus) and general (superior and middle temporal gyri) visuospatial impairment. This study highlights the prevalence and diversity of visuospatial impairments that can occur following stroke.

## Teaser

Visuospatial neglect is a disorder diagnosed with clinical examinations that cannot capture processing speed. Park *et al.* examined reaching movements in 265 individuals with stroke and found almost half had reaction time impairments limited to a specific direction or spanning all directions. Reaction time impairments were associated with specific lesions.

## Graphical Abstract

**Graphical Abstract:**
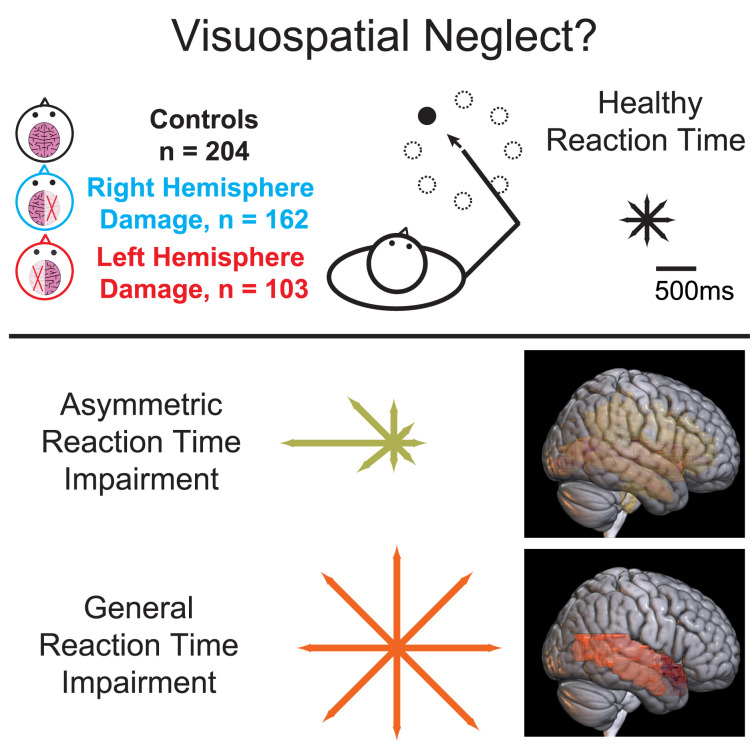

## Introduction

Visuospatial neglect is a common disorder seen after stroke although its incidence across studies varies widely from as little as 8% to as high as 80%.^1‐3^ It is commonly characterized by an inability to attend to visual stimuli in the contralesional workspace with a higher prevalence and severity for individuals with right hemisphere damage (RHD) than left hemisphere damage (LHD).^4^ There have been many tests developed to quantify visuospatial neglect with the majority based on scores determined by pen and paper examinations. The focus of these tests is to assess whether individuals with stroke exhibit asymmetry in visual processing by scanning various stimuli on a sheet of paper. Common assessments include cancellation tests,^5‐8^ line bisection tests^4,9^ and figure drawing/copying,^10‐12^ which have been shown to be both valid and reliable measures.^13‐17^ The amount of different tests developed to assess neglect may be attributed to the many proposed subtypes of the disorder.^18‐21^

Critically, these tests have no time limits and are static examinations and thus cannot quantify potentially important impairments in responding to environmental cues. Reaction time (RT) is a particularly useful measure as it can gauge how quickly visual information can be processed to initiate a motor action. Healthy individuals can initiate a reach in a few hundred milliseconds,^22,23^ but studies have shown that individuals with visuospatial neglect can have delayed RT in their affected visual field even with their ipsilesional arm.^24‐26^ Heilman *et al.*^24^ reported that individuals with RHD and visuospatial neglect displayed longer RT when reaching towards the left side of space as compared to the right, and this has been corroborated by others.^27‐31^ There have been few studies examining RT impairment for individuals with LHD despite clear impairments identified for these individuals.^32,33^ There is also evidence to suggest that non-lateralized RT impairment in individuals with visuospatial neglect may exacerbate lateralized impairments.^34,35^ These impairments become increasingly important as delays in RT for individuals with stroke have been reported to increase risk of injury^36^ and reduce quality of life.^37^

Here, we develop techniques to quantify the breadth and directionality of reaching RTs in different spatial directions along the horizontal plane. Our study adapts techniques used in the analysis of circular data to identify any directionality in RTs,^38,39^ commonly used to quantify neural activity.^40,41^ Based on previous findings, we expect to identify leftward RT impairment for individuals with RHD as well as rightward impairment for individuals with LHD but also hypothesize that some individuals may have impairments when reaching in all directions.^35^

## Materials and methods

### Subjects

Participants with stroke were recruited from the stroke units in Calgary, Alberta, at the Dr. Vernon Fanning Centre and Foothills Medical Centre and in Kingston, Ontario, at the St. Mary’s of the Lake Hospital and Providence Care Hospital. Patients were recruited if they were 18 years and older with a first time reported ischaemic or haemorrhagic stroke. Patients were excluded if they had a previous stroke, non-stroke–related neurological disease (e.g. Parkinson’s disease) and ongoing upper extremity musculoskeletal injury or were unable to understand task instructions.

Neurologically healthy control participants were recruited from the communities in Kingston, Ontario and Calgary, Alberta. Exclusionary criteria were the same as for participants with stroke. This study was approved by the University of Calgary and Queen’s University Research Ethics Boards, and all participants provided informed consent.

### Clinical measures

Participants with stroke were assessed by trained clinicians using a number of standard clinical assessment tools. First, patients were assessed using the Behavioral Inattention Test (BIT) to measure visuospatial inattention.^42^ The BIT was used to determine visuospatial neglect as it is a common clinical tool that comprises a battery of tests that largely focuses on spatial impairment. The conventional BIT consists of six pen and paper tests that look to assess different forms of neglect. Individuals who score <130 out of a possible 146 on this measure were identified as having visuospatial neglect.^42^ The BIT was also assessed on healthy controls to compare control performance in our communities with the control performance in the original paper.^42^ Visual field deficits were assessed using the confrontation technique. Briefly, the confrontation technique is a clinician-observed measure whereby participants (with one eye blocked) are tasked with following the clinician’s hand moving to one of four quadrants of the visual field. They are then asked how many fingers are being held up and this sequence is repeated for the other three quadrants and then the other eye.^43^ Independence for activities of daily living (ADL) was examined with the Functional Independence Measure (FIM).^44^ This is a measure of 18 activities from eating and toileting to memory and problem-solving where each activity is graded from 1 (total assistance/not testable) to 7 (complete independence). Physical impairment in both arms was assessed using the Chedoke-McMaster Stroke Assessment Impairment Inventory (CMSA) characterizing arm performance where scores range from 1 (no functional abilities) to 7 (full ability to perform all actions).^45^ Cognition was screened with the Montreal Cognitive Assessment (MoCA) where scores (0–30) below 27 suggest the presence of impaired cognitive function.^46^

### Robotic setup

Reaching performance was assessed using the Kinarm Exoskeleton Lab (Kinarm, Kingston, Canada).^47^ Participants were seated in a height-adjusted chair that provides truncal support, if required. A robotic exoskeleton supported the upper arms and forearm/hands to allow free movement of both arms in the horizontal plane. A virtual reality system was aligned with the workspace of the arms and displayed visual feedback of spatial goals and the position of the index finger (Supplementary Fig. 1). Direct vision of the arms was occluded by a physical barrier. Participants were examined on each arm separately, and the order of examination was random.

### Robotic assessment

Visuospatial performance was assessed using an eight-target centre-out visually guided reaching (VGR) task (Supplementary Fig. 1). The task has been described in detail previously.^22^ Briefly, participants were required to start each trial by moving their index finger (represented as a 0.8 cm diameter white circle) to a central, red start target (diameter = 2 cm) positioned in front of the arm at approximately 90° elbow flexion and 30° shoulder flexion. After a random time (1250–1750 ms), one of eight peripheral red targets was illuminated and the subject was required to reach quickly and accurately to this second target. The red start target was turned off as soon as subject’s index finger left the start target. Peripheral targets were distributed uniformly 10 cm away from the central start target and appeared in a pseudorandom order with each target appearing eight times for a total of 64 reaches. There were also 16 interleaved trials in which no peripheral target was illuminated, and participants were required to maintain their hand at the start target. This task was performed with both arms, and the initial arm assessed was randomly selected.

### Data analysis

Movement initiation was identified using an algorithm that was adjusted for each participant based on their range of hand speeds during the postural hold period (for full details, see website for Kinarm Standard Test Summary, kinarm.com).^22^ RT was then defined as the time interval between peripheral target illumination and movement onset. If the participant remained at the start target for the entire trial (i.e. did not react to the peripheral target), then RT was set to the maximal allotted time for reaching (3000 ms). For each movement direction, we calculated the mean RT for all trials.

The next step of our analysis transformed RT into standardized units that factored out the influence of age, sex and handedness.^48‐50^ As well, reaching performance varies across movement direction due to the influence of limb biomechanics.^51^ We considered the influence of these factors by assessing reaching performance in 204 neurologically healthy individuals. This group included males (*n* = 102) and females with an age range from 18 to 83. For each spatial direction, RT measures were transformed into a standard normal distribution using Box-Cox equations^52^ and linear regressions to account for age, sex and handedness. This distribution was checked for normality with a Shapiro–Wilk test. Healthy individuals whose *Z*-score was greater than 3.29 or less than −3.29 were then removed as outliers, and the entire process was repeated up to three times or until there were no further outliers. This model was then used to transform the RT for each individual with stroke into a measure in *Z*-scores (see Kinarm Standard Test Summary, kinarm.com).^53^

### Measure analysis

**Figure 1:**
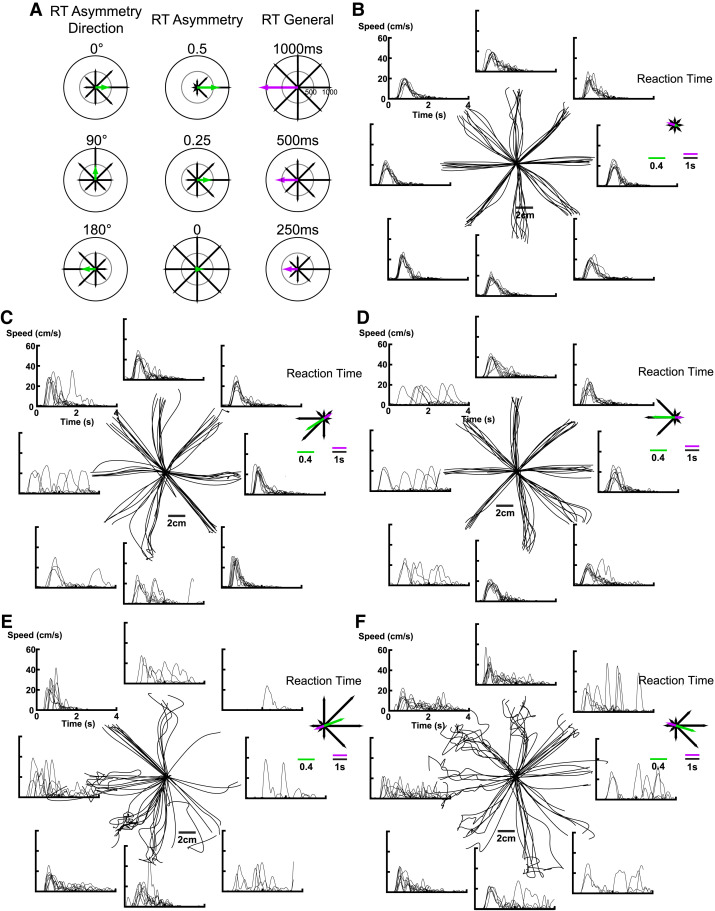
**Hand paths, hand speeds and RT of exemplar participants.** (**A**) Didactic polar plots of RT parameters. Each plot illustrates average RT values as black arrows for each of the directions of reach in the VGR task. The left column displays three plots demonstrating the RT asymmetry direction at different angles. The *middle* column highlights three examples of different RT asymmetry (green arrow) possible based on the RT (black arrows). The right column displays three values of RT general (purple arrow) when given different arrays of RT. Note that RT general values are in milliseconds for didactic purposes, but impairments in Table 2 were defined from the transformed data that corrected for age, sex, handedness and target direction. (**B**) The hand paths and corresponding hand speeds of the right hand of a 57-year-old right-handed female control participant. The panel on the right displays the RT and resultant RT asymmetry direction, RT asymmetry and RT general for this participant. (**C, D**) The hand paths and speeds of the left and right hand, respectively, of a 65-year-old right-handed female individual with RHD. This individual had a BIT score of 139, CMSA arm scores of 7 and MoCA of 28. No visual field deficits were evident based on clinical examination. (**E, F**) Hand paths and speeds of the left and right hands, respectively, of an 85-year-old right-handed individual with LHD. This participant had a BIT score of 144, CMSA arm scores of 7 and MoCA of 25. No visual field deficits were evident at examination.

We developed three parameters, RT asymmetry direction, RT asymmetry and RT general, to find a potential bias in the spread of multidirectional data (Fig. 1A).^38,39^ Similar measures have been used previously to quantify neural activity in awake behaving monkeys performing centre-out reaching.^40,41,54^ The first parameter, RT asymmetry direction, denotes the spatial direction in which participants performed worse (longest RT) compared to other directions. The second parameter, RT asymmetry, quantifies the variation in RT across directions. These two parameters work in conjunction to describe the direction (RT asymmetry direction) and spread of the data (RT asymmetry) towards a specific spatial direction. Both calculations require values to all be positive. Therefore, RT *Z*-scores were converted into positive values using the complementary error function.^55^ This process converts the two-sided *Z*-score of positive and negative values, where best performance is centred on 0, to a one-sided *Z*-score of positive values, where best performance is grounded at 0.^53^

The RT asymmetry direction $(\theta_{\mathrm{RT}})\;$ and RT asymmetry (*d*_RT_) were calculated using the following steps:

- Calculate the sum of the horizontal components of each RT (RT_HC_) based on their respective movement direction angle ($\theta_{i}$ where 0° is the target to the right) and divide them by the sum of RT in all directions (*n* = 8) (RT_Total_)
(1) $$RT_{\mathrm{HC}}=\frac{\left(\sum_{i=1}^{n}RT_{i}\times \cos\;\theta_{i}\right)}{RT_{\mathrm{Total}}}$$
- Repeat this process for the vertical components (RT_VC_)
(2) $$RT_{\mathrm{VC}}=\frac{\left(\sum_{i=1}^{n}RT_{i}\times \sin\;\theta_{i}\right)}{RT_{\mathrm{Total}}}$$
- Calculate the hypotenuse (*d*_RT_) and corresponding angle (*θ*_RT_) between the horizontal and vertical components
(3) $$\theta_{\mathrm{RT}}=\tan^{-1}\frac{RT_{\mathrm{VC}}}{RT_{\mathrm{HC}}}$$
(4) $$d_{\text{RT}}=C_{\text{f}}\sqrt{{\text{RT}}_{\text{HC}}{}^{2}+{\text{RT}}_{\text{VC}}{}^{2}}$$
  where $\theta_{\mathrm{RT}}$ is the direction with the longest RT (RT asymmetry direction), *d*_RT_ is the size of asymmetry in the RT across directions (RT asymmetry) and *C*_f_ is a correction factor related to the number of spatial directions sampled in the dataset (*n* = 8 for the present study).

The third parameter was quantified by RT general (Gen_RT_) for each subject. This was assumed to be associated with targets in the direction opposite to $\theta_{\mathrm{RT}}$ and calculated with the following procedure (Fig. 1B):

- Identify the target direction that is closest to $\theta_{\mathrm{RT}}$ ±180.
- Quantify Gen_RT_ by averaging the RT of that target with the two adjacent targets.

RT asymmetry was sensitive to the relative range of values across spatial directions. That is, after normalizing the RT and converting to *Z*-scores, an individual with very short RTs (and thus values near 0) could have a large RT asymmetry even though there was a relatively small change in values across directions. This sensitivity to small variations reduced as the range of values moved away from 0, and thus, quantifying normalized RT asymmetry required an offset. In order to find an optimal offset, we systematically added offsets, *S*, in increments of 0.25 and then recalculated RT asymmetry direction and RT asymmetry for both the controls and individuals with stroke. Our objective was to select the smallest offset that identified the maximal number of individuals with stroke as different from controls (outside the 95th percentile of healthy controls). In general, larger values of *S* led to more stroke participants being identified outside the range of healthy controls but were asymptotic for values above 5. Thus, we used an offset of 5 to calculate RT asymmetry direction and RT asymmetry.

### Image acquisition

Images were collected in accordance with the clinical acute stroke imaging protocols at Foothills Medical Centre. Where possible, MRI was collected and included fluid-attenuated inversion recovery (FLAIR), diffusion-weighted imaging (DWI) and apparent diffusion coefficient (ADC) sequences. In cases where MRI was not possible, standardized CT protocols were used. MRI was collected using a General Electric 1.5 or 3.0 T scanner. CT imaging was collected using either of three General Electric CT scanners or a Siemens system scanner.

### Lesion delineation and registration

Lesions were all marked on the participants’ anatomical images by trained markers and verified by a stroke neurologist. For MRI scans, lesions were marked on the FLAIR image, using the DWI and ADC to guide marking where possible. Lesion markings were registered to a standard Montreal Neurologic Institute (MNI) template using a clinical toolbox (nitrc.org/projects/clinicaltbx)^56^ in SPM12 (fil.ion.ucl.ac.uk/spm/software/spm12) and visually checked for accuracy.

### Statistical region of interest mapping

Statistical region of interest (sROI) mapping was performed on each RT parameter. First, each individual’s registered lesion was parcellated, and the extent of damage to each of the 150 regions was calculated. The 150 regions were determined by the automated anatomical labelling (AAL) and Catani (natbrainlab.com/) atlases.^57,58^ To determine the relationship between the extent of damage to each region of these atlases and scores on each behavioural parameter (RT asymmetry and RT general), a whole-brain analysis was performed using NiiStat (nitrc.org/projects/niistat/). We only analysed regions where at least five people had lesions within that region. The data were permuted 4000 times to control for family-wise error, and a significance threshold of *P* < 0.05 was used for the sROI analysis.

### Statistical analysis

Spearman’s correlation coefficients were used to compare clinical scores and RT parameters (corrected for age, sex, handedness and direction; see ‘Data analysis’). Significant difference between two groups of participants (e.g. individuals with RHD and LHD, and male and female) was performed by comparing the two distributions with a Kolmogorov–Smirnoff test. Some of our analyses were performed on circular data, and, as such, commonly used methods for determining correlations are not suitable. We used Rayleigh’s test^38,39^ to identify whether the observed RTs were unimodally ‘tuned’ across the eight spatial directions with slower RTs in one spatial direction and faster RTs in the opposite direction. Finally, we used the Watson–Williams test to determine whether the directional tuning was different between two sets of circular data (i.e. RT directional tuning for left and right arms across all individuals with stroke). The Watson–Williams test was only reported if the data passed the assumptions of the test. All statistical calculations were performed with MATLAB Statistics and Machine Learning Toolbox (Mathworks, R2018b) and the toolbox for circular statistics with MATLAB.^39^

## Results

Table 1 displays demographic information and clinical scores for the 265 individuals with stroke and 204 controls (female = 102). Our cohort included a greater number of individuals with stroke that were right hemisphere damaged (RHD = 162) as compared to left hemisphere damaged (LHD = 103). One potential reason for a bias in the number of RHD over LHD individuals may be due to a greater language impairment associated with left hemisphere damage, and thus, these individuals were not eligible to be recruited for this study.^59,60^ Individuals who were unable to perform at least 16 trials (two trials in each direction) with their ipsi- and contralesional arms had data for that respective arm removed. In total, 35 individuals with RHD and 22 with LHD were unable to complete the minimum number of trials using their contralesional arm. All individuals with RHD and all but one with LHD were able to complete the minimum number of trials using their ipsilesional arm. Normalization of healthy controls’ RT removed three outliers.

**Table 1:** Demographic and clinical data of controls and stroke

	Controls (*n* = 204)	Individuals with RHD (*n* = 162)	Individuals with LHD (*n* = 103)
AGE (RANGE)	50 (18–83)	63 (28–89)	61 (18–91)
SEX (M/F)	102/102	112/50	65/38
HANDEDNESS (L/R/A)	10/194/0	6/155/1	10/93/0
STROKE TYPE (I/H/U)		136/21/5	89/12/2
DAYS SINCE STROKE (RANGE)		12 (1–59)	11 (2–34)
VISUAL FIELD CUTS		14	1
FIM (RANGE)		96 (35–126)	96 (38–126)
CMSA CONTRALESIONAL ARM (1/2/3/4/5/6/7)		9/19/19/10/35/29/40	10/8/16/7/20/15/26
CMSA IPSILESIONAL ARM		0/0/0/0/5/25/131	0/0/0/0/0/13/89
CMSA CONTRALESIONAL HAND		15/8/12/11/48/39/27	14/6/10/11/23/17/21
CMSA IPSILESIONAL HAND		0/0/0/0/3/47/110	0/0/0/0/3/25/74
MOCA (RANGE)		24 (11–30)	22 (7–30)
BIT (RANGE)	144 (135–146)^a^	133 (58–146)	139 (105–146)
NO. OF FAILED BIT		36	9
STROKE LOCATION (c/sc/c + SC/CE/BR/CE + BR/U)		39/45/53/1/12/0/12	19/39/26/2/11/1/5

Demographic information for control and individuals with stroke along with relevant clinical data. Age, days since stroke, FIM, MoCA and BIT are displayed as mean along with the range. Sex, handedness, stroke type, CMSA and stroke location are shown as values split according to the subtypes identified as follows: sex (male/female); handedness (left/right/ambidextrous); stroke type (ischaemic/haemorrhagic/unknown); CMSA scores of (1/2/3/4/5/6/7); stroke location (cortical/subcortical/cortical + subcortical/cerebellar/brainstem/cerebellar + brainstem/unknown).

a The BIT was assessed in a subset of only 73 controls.

There was a broad range of BIT scores for individuals with stroke, ranging from 146 to 58. Individuals with RHD generally performed worse on the BIT as compared to the individuals with LHD (Kolmogorov–Smirnov test, *P* < 0.05), and there was a greater percentage of individuals with RHD identified as having visuospatial neglect, receiving scores <130 (22% for RHD and 9% for LHD). It is interesting to note that control participants made few errors, and we found that 95% of these individuals scored above 140. This distribution was similar to the one observed in the control participants of the original paper^42^ (*n* = 50), and thus, the BIT was only assessed in 73 controls. A large portion of stroke participants scored between 130 and 140 (30%) and, thus, were not traditionally defined as having visuospatial neglect despite performing worse than 95% of healthy controls. Visual field deficits were evident for 14 individuals with RHD and only 1 individual with LHD.

### Individual reaching performance

Figure 1 displays hand trajectories and hand speeds for movements to the eight spatial targets. We present didactic polar plots with each set of black arrows denoting RT in the eight directions of reach for the VGR task (Fig. 1A). RT asymmetry direction (left column) denotes the direction with the slowest RT displayed here to the right (0°), away (90°) or the left (180°), top, middle, and bottom, respectively. RT asymmetry (green arrows in the middle column) reflects the amount RT varies across movement directions from 0.5 to 0. RT general (purple arrows in the right column) captures the fastest RT, assessed in the direction opposite to RT asymmetry direction.

Figure 1B displays reaching performance of the right-dominant arm of a control participant. Hand paths were relatively straight from the central start target to the eight peripheral targets. Hand speeds displayed typical bell-shaped profiles with an average RT of 389 ms across all directions. The inset panel on the top right displays a polar plot of RT, as demonstrated by Fig. 1A, showing the eight arrows with similar lengths denoting that the RT for this individual was similar across movement directions. The green arrow denotes a small RT asymmetry of 0.023 oriented to 342°. RT opposite to the RT asymmetry was calculated to determine general RT performance. This is represented by a purple arrow with an RT general of 377 ms (similar length to the black arrows).

Figure 1C and D depict contralesional and ipsilesional arm reaching, respectively, for an individual with RHD that exhibited longer RT for leftward movements (BIT score = 139). For the contralesional arm (Fig. 1C), movements to the top right had relatively straight hand paths and speed profiles with average RT similar to controls. In contrast, movement initiation toward the bottom left and surrounding targets were inconsistent with some trials resulting in no motor responses and, thus, increased mean RT. The length of the black arrows in the top right panel denotes the variation in RT across directions. The green arrow depicts a large RT asymmetry of 0.43 oriented at 213°. The purple arrow represents a RT general of 444 ms. The ipsilesional arm (Fig. 1D) performed similar to the contralesional arm with a slight decrease in the variability in hand trajectories from trial to trial. RT was again substantially slower for movements to the left leading to a RT asymmetry of 0.47 at 179°. Of interest, the RT asymmetry direction of the contralesional arm was oriented to 213° but was 179° for the ipsilesional arm. This produced a clockwise shift in RT asymmetry direction between arms of ∼34°. The RT general for the ipsilesional arm of 379 ms was shorter than the contralesional arm.

Figure 1E and F depict reaching of an individual with LHD and clear difficulties reaching to rightward targets with both arms (BIT score = 144). Using their ipsilesional arm (Fig. 1E), the individual was able to initiate leftward reaches but had difficulty completing the reach to the spatial goal. In contrast, movement initiation to the right was inconsistent leading to a long average RT depicted by the black arrows representing RT in the top right panel. This resulted in a green arrow depicting a RT asymmetry of 0.42 at a direction of 21°. The purple arrow depicting RT general was 526 ms. The contralesional arm, shown in Fig. 1F, displayed similar impairments as the ipsilesional arm. For leftward movements, the individual, again, could initiate the reach but had difficulty completing the last portion. Rightward movements had inconsistent RT across trials which increased the average RT. The resultant RT asymmetry was 0.41 and RT general was 496 ms. Interestingly, the difference in orientation of RT asymmetry direction between the left and right arms was ∼38° clockwise (21° for the ipsilesional arm and 343° for the contralesional arm).

We performed a Kolmogorov–Smirnoff test between control participants and individuals with stroke and identified a significant difference between these two groups for both RT asymmetry (*P* < 0.001) and RT general (*P* < 0.001). Below, we separate the analysis of individuals with RHD and LHD given the expected differences in their performance.^61‐63^

### Individuals with right hemisphere damage

Table 2 displays individuals with stroke impaired for RT asymmetry, RT general or both. We found that a large portion of individuals with RHD were impaired in either RT asymmetry or RT general with 64% impaired in their contralesional arm and 48% impaired in their ipsilesional arm. RT asymmetry impairment was similar for both arms of individuals with RHD where 35% were impaired in the contralesional and 34% in the ipsilesional arm. Impairments in RT general tended to be greater in the contralesional arm compared to the ipsilesional arm (44% versus 32%). Of note is the portion of individuals with RHD who presented with impaired RT asymmetry and a similar portion with impaired RT general (34% and 32%, respectively) in their ipsilesional arm. Examining the proportion impaired by those who passed and failed the BIT highlighted some interesting results. The majority of individuals that failed the BIT also had impaired RT performance (91% in the contralesional arm and 94% in the ipsilesional arm). However, a large proportion who passed the BIT also displayed impaired RT performance (58% in the contralesional arm and 35% in the ipsilesional arm). There was a small proportion of individuals who failed the BIT and were not impaired in RT (9% in the contralesional arm and 6% in the ipsilesional arm).

**Table 2:** Individuals with RHD and LHD normalized VGR performance displaying RT impairments

	RT asymmetry and/or general impaired	RT asymmetry impaired	RT general impaired	No RT impairment		RT asymmetry and/or general impaired	RT asymmetry impaired	RT general impaired	No RT impairment
Individuals with RHD									
	Contralesional arm (*n* = 127)					Ipsilesional arm (*n* = 162)			
Passed BIT (*n* = 104)	60 (58%)	26 (25%)	43 (41%)	44 (42%)	Passed BIT (*n* = 126)	44 (35%)	28 (22%)	29 (23%)	82 (65%)
Failed BIT (*n* = 23)	21 (91%)	19 (83%)	13 (57%)	2 (9%)	Failed BIT (*n* = 36)	34 (94%)	27 (75%)	23 (64%)	2 (6%)
All BIT (*n* = 127)	81 (64%)	45 (35%)	56 (44%)	46 (36%)	All BIT (*n* = 162)	78 (48%)	55 (34%)	52 (32%)	84 (52%)
Individuals with LHD									
	Ipsilesional arm (*n* = 103)					Contralesional arm (*n* = 81)			
Passed BIT (*n* = 94)	26 (28%)	20 (21%)	10 (11%)	68 (72%)	Passed BIT (*n* = 78)	37 (47%)	14 (18%)	27 (35%)	41 (53%)
Failed BIT (*n* = 9)	5 (56%)	5 (56%)	2 (22%)	4 (44%)	Failed BIT (*n* = 3)	1 (33%)	1 (33%)	0 (0%)	2 (67%)
All BIT (*n* = 103)	31 (30%)	25 (24%)	12 (12%)	72 (70%)	All BIT (*n* = 81)	38 (47%)	15 (19%)	27 (33%)	43 (53%)

Number and percentage of individuals with stroke impaired in RT. Individuals with stroke are separated into RHD and LHD groups with separate analysis of the contra- and ipsilesional arms. Impairment in RT is defined as having RT greater than 95% of controls and is separated into RT asymmetry and/or general, RT asymmetry alone, RT general alone, or no RT impairment. Further, individuals with stroke are separated into categories based on whether they had passed (>129) or failed (<130) the BIT.

**Figure 2:**
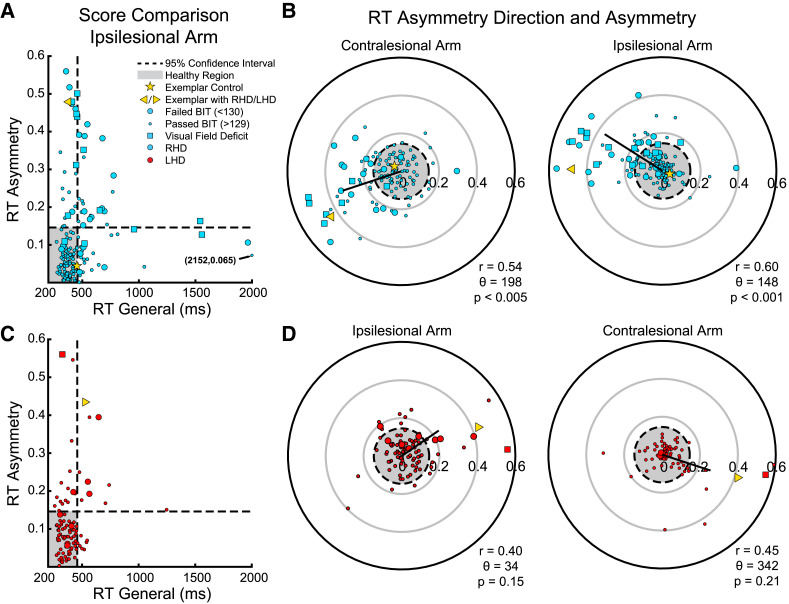
**RT asymmetry direction, asymmetry and general for all individuals with stroke.** (**A**) The RT asymmetry plotted against the RT general of the ipsilesional arm of individuals with RHD. The yellow left-directed triangle represents the exemplar individual with RHD, and the yellow star represents the exemplar control both displayed in Fig. 1. Each blue circle represents an individual with RHD where the circle sizes denote those who passed the BIT (small circles, score <130) or failed the BIT (large circles, score ≥130). Individuals with visual field deficits are highlighted as squares. Dashed lines denote the 95% CI of healthy controls where the grey zone highlights the ‘healthy’ region. (**B**) RT asymmetry direction plotted against RT asymmetry for the contra- and ipsilesional arm of individuals with RHD. The left polar plot displays RT performance for the contralesional arm of individuals with RHD and the previous exemplar control and stroke participant as a yellow star and triangle, respectively. Circular correlations of all individuals with RHD are plotted as the black arrow (Rayleigh’s test, *Z* = 5.9). The right polar plot displays the ipsilesional arm of individuals with RHD in the same format as the left plot (Rayleigh’s test, *Z* = 7.9). (**C**) RT asymmetry plotted against RT general of the ipsilesional arm of individuals with LHD in the same format as **A**. The individual with LHD from Fig. 1 is displayed as the right-directed yellow triangle. (**D**) RT asymmetry direction plotted against RT asymmetry for the ipsi (Rayleigh’s test, *Z* = 1.5) and contralesional arms (Rayleigh’s test, *Z* = 2.1) of individuals with LHD in the same format as **C**.

Figure 2A displays RT asymmetry versus RT general in the ipsilesional arm of individuals with RHD. The horizontal and vertical dashed lines represent the 95% confidence interval (CI) of control RT asymmetry and RT general, respectively. Individuals above the horizontal or right of the vertical line were deemed as impaired. Of note is the diversity of impairments across the cohort and the natural trade-off as very large values for RT asymmetry tend to have small values for RT general and vice versa. Further, individuals with visual field deficits demonstrated impairments in both RT measures rather than only RT asymmetry.

Figure 2B displays polar plots of RT asymmetry for individuals with RHD in both arms. The dashed black circle (radius = 0.15) represents the outside boundary for healthy controls, and individuals with stroke outside of this circle were identified as having an impaired RT asymmetry. Most impaired individuals with RHD were slower to initiate reaches for leftward targets for both arms (RT asymmetry direction 198° and 148°, respectively) which, interestingly, led to a systemic clockwise rotation of ∼50° between the arms (Watson–Williams test, *P* < 0.001).

RT measures were compared to clinical scores for individuals with RHD in their ipsilesional arm. There is a moderate negative correlation between RT asymmetry and the BIT (*r* = −0.49, *P* < 0.001) and RT general with the BIT (*r* = −0.38, *P* < 0.001). We found non-significant correlations between RT asymmetry and FIM (*r* = −0.081, *P* = 0.31) and a weak significant correlation between RT general and FIM (*r* = −0.16, *P* < 0.05).

### Individuals with left hemisphere damage

ndividuals with LHD were overall less impaired than individuals with RHD (see Table 2). RT impairment for individuals with LHD was evident for 30% in the ipsilesional arm and 47% in the contralesional arm. Importantly, RT asymmetry impairment was more prevalent than RT general impairment in the ipsilesional arm (24% and 12%, respectively). This pattern was found regardless of BIT score potentially due to the low numbers of individuals who failed the BIT (*n* = 9).

Figure 2C shows the comparison between RT asymmetry and RT general in the ipsilesional arm of individuals with LHD. As mentioned previously, there were less individuals with LHD impaired than individuals with RHD, but nonetheless, we identified individuals with LHD that passed the BIT (small circles) and had RT impairment. Of note is the small number of individuals with RT general impairment.

Figure 2D depicts the RT asymmetry and RT asymmetry direction for both arms of individuals with LHD. The systemic shift seen in individuals with RHD was also seen for individuals with LHD. The ipsilesional arm of individuals with LHD was impaired towards 34°, and the contralesional arm was impaired towards 342° resulting in a systemic shift of approximately 52° (Watson–Williams test, *P* < 0.005).

The correlations between RT and clinical scores were moderate at best. There was a moderate correlation between RT asymmetry and BIT for individuals with LHD (*r* = −0.37, *P* < 0.001). The correlation between RT general and BIT was not significant (*r* = −0.15, *P* = 0.14). RT asymmetry and FIM correlations were not significant (*r* = −0.11, *P* = 0.31), and RT general and FIM scores were weakly correlated (*r* = −0.26, *P* < 0.01).

### Statistical region of interest analysis

**Figure 3:**
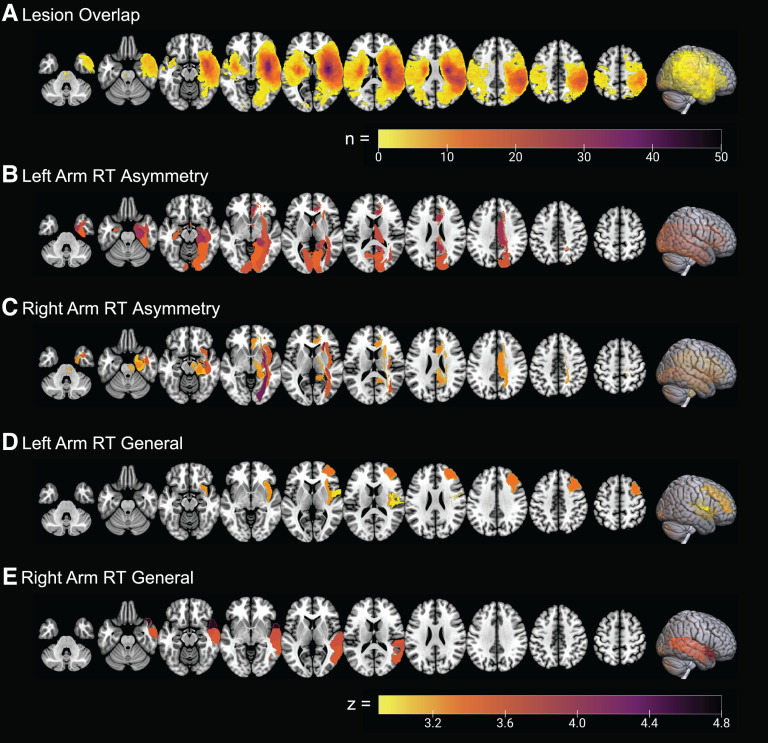
**Lesion overlap maps of all stroke individuals.** (**A**) Overlap of lesion location for all individuals with stroke (*n* = 201). (**B, C**) sROI analysis lesion location for those impaired in RT asymmetry of the left and right arms, respectively. Note that RT impairments were defined based on transformed data. (**D, E**) sROI maps for those impaired in RT general in the left and right arms, respectively.

MRI was collected for 201 stroke individuals, which included FLAIR, DWI and ADC sequences. MRI scans were not accessible in 42 patients, and CT scans were used instead. Table 3 displays the *Z*-scores determined from the statistical comparison between the lesion location and RT parameters. The sROI analysis yielded significant relationships with RT measures, largely in the right hemisphere (Table 3 and Fig. 3). RT asymmetry for the left arm identified 15 regions, including regions within the occipital and temporal lobes, in addition to regions of the limbic system. The majority of these regions resided in the right hemisphere but a few regions in the left hemisphere were identified (hippocampus and calcarine sulcus). RT asymmetry for the right arm identified eight regions, including the insula, parts of the occipital lobe and white matter tracts stemming from the occipital lobe [inferior frontal–occipital fasciculus (IFOF) and inferior longitudinal fasciculus (ILF)] in the right hemisphere. RT general for the left arm identified four regions in the right hemisphere, including the insula, rolandic operculum and anterior segment. Finally, RT general for the right arm identified three regions within the right temporal lobe [of note the superior and middle temporal gyri (STG and MTG)].

**Table 3:** Statistical regions of interest determined by normalized VGR performance of the stroke population

Region	*Z*-score
Left arm RT asymmetry	
Right hippocampus	4.1
Right parahippocampal gyrus	4
Right cingulum	4
Right fornix	3.8
Right inferior longitudinal fasciculus	3.8
Right amygdala	3.7
Right optic radiations	3.7
Left calcarine sulcus	3.6
Right cuneus	3.6
Right superior occipital lobe	3.6
Right inferior frontal–occipital fasciculus	3.6
Left hippocampus	3.5
Right calcarine sulcus	3.5
Right lingual gyrus	3.4
Right fusiform gyrus	3.4
Right arm RT asymmetry	
Right inferior frontal–occipital fasciculus	4.4
Right optic radiations	3.9
Right insula	3.6
Right inferior longitudinal fasciculus	3.6
Right cingulum	3.3
Right hippocampus	3.2
Right internal capsule	3.2
Right parahippocampal gyrus	3.1
Left arm RT general	
Right middle frontal gyrus	3.3
Right insula	3.2
Right anterior segment	3.1
Right rolandic operculum	3
Right arm RT general	
Right superior temporal pole	4.7
Right superior temporal gyrus	3.7
Right middle temporal gyrus	3.6

Lesion locations identified as significantly associated with impaired RT asymmetry and RT general using the left and right arms. *Z*-scores quantify the statistical association between the extent of damage to each region and each parameter (only presenting those regions which survived permutation testing). Comparisons were made using a critical one-sided *P*-value of 0.05 and 4000 permutations (one sided assumed as cortical damage is associated with worse behavioural outcomes).

### Sex differences in reaction time

Average RT was examined in our healthy controls and individuals with stroke. RTs of male controls (mean ± SD, 374 ± 90 ms) were similar with females (378 ± 101 ms), and this was not significantly different (Kolmogorov–Smirnov test, *P* = 0.60). Analysis of RT measures did not identify significant correlation in the RT asymmetry in either males (*r* = 0.12, *θ* = 29°, *P* = 0.76) or females (*r* = 0.17, *θ* = 81°, *P* = 0.74), but the circular means were significantly different (*P <* 0.001). For individuals with stroke, RTs of males (627 ± 547 ms) were slower than females (561 ± 310 ms), but the distribution of means was not significantly different (Kolmogorov–Smirnov test, *P* = 0.075). RT asymmetry was not significantly correlated in males with stroke (*r* = 0.21, *θ* = °, *P* = 0.07) or females with stroke (*r* = 0.16, *θ* = °, *P* = 0.50), but circular means were significantly different from each other (Watson–Williams test, *P* < 0.001).

## Discussion

The present study examined reaching performance in a large cohort of individuals with RHD as well as individuals with LHD. We found that a large proportion of individuals had RT impairments with their ipsilesional arm (48% for RHD and 30% for LHD). RT impairments could be limited to a limited spatial region or span all spatial directions. We also observed that the directionality of RT impairments rotated clockwise when reaching with the left and right arms regardless of damaged hemisphere. While many of those identified with visuospatial neglect based on BIT scores were identified with RT impairments, a substantive proportion of those that passed the BIT also had RT impairments. Imaging data using sROI analysis identified distinct lesion sites; of note are the insula, IFOF and ILF for those with directional RT impairment and the STG and MTG for individuals with general RT impairment.

### Spatial features of reaction time impairments

Our study corroborates previous findings demonstrating individuals with RHD commonly have RT impairments when reaching to the left.^26,64,65^ However, we also demonstrated that almost an equal number had RT impairments in all spatial directions. In contrast, we found that individuals with LHD were approximately twice as likely to have RT asymmetry impairment compared to impairments in all spatial directions (24% versus 12%, respectively). These trends support the common view that the left hemisphere tends to be associated with visuospatial control for the right side of space, whereas the right hemisphere tends to be associated with both the left and right hemispace.^61,66‐70^

Previous studies exploring RT impairments for individuals with stroke commonly quantified visuomotor responses in two spatial directions, to the left or right.^26,29,71^ Our approach of assessing reaching in eight directions allowed us to quantify the directionality and spatial breadth of RT impairments. While many individuals with stroke displayed directional impairments roughly directed to the left or right, there were many individuals with RT impairments directed away from or even towards themselves that would not be captured. Early studies of visuospatial neglect highlighted attentional impairments observed in the vertical direction^72,73^ or both vertical and horizontal dimensions.^27,74‐77^ Studies have also pointed to an influence of stimulus depth and neglect.^72,78^ Taken together, RT impairments are highly variable across individuals and can span a small portion of the three-dimensional workspace or all spatial directions.

Not surprisingly, most studies only examine RT for the ipsilesional arm to attempt to reduce the influence of motor impairments unrelated to visuospatial neglect.^79^ Even though motor-related impairments may influence measures of RT, our results highlight two interesting characteristics of RT impairments only identified with the examination of both arms. We noted a general rotation in the direction of RT asymmetry across the arms with a ∼50° clockwise rotation from the left to right arms across the entire stroke cohort. This first suggests that impairment commonly associated with the contralesional arm may not be only due to motor impairment but is also influenced by RT impairment. Second, in our experimental layout, the starting location of the hand was roughly aligned with the respective shoulder (30° forward shoulder flexion and 90° elbow flexion). Thus, the head rotates ∼50° when orienting gaze at the central start position for the left versus right arms, suggesting RT impairments reflect an egocentric frame of reference.^80,81^

### Visuospatial neglect and reaction time impairments

Previous studies that explore RT impairments following stroke commonly group individuals into neglect and non-neglect based on the presence of visuospatial neglect using clinical tools such as the BIT. These studies focus on individuals with RHD and find that the neglect group has longer RT than healthy controls^24‐26,31,71^ or the non-neglect group.^24,71,82^ This grouping of neglect and non-neglect can be problematic as it assumes that visuospatial neglect is a disorder defined by impairment on pen and paper examinations rather than real-world interactions which require complex visuospatial ability.

Our approach was to explore RT impairments in a large cohort of individuals with RHD and LHD, of which the latter are rarely assessed.^31,33^ Critically, we identified the bounds of healthy control performance and carefully corrected for RT variation due to the impact of limb mechanics and other factors. This allowed us to identify whether each individual with stroke was impaired rather than simply quantifying group effects. Not surprisingly, individuals that failed the BIT commonly displayed RT impairments reflecting the fact that both assess the ability to identify and interact with the visuospatial environment. However, we also found individuals who failed the BIT but did not have RT impairment and vice versa.

An important question is why there is a discrepancy between clinical tools designed to diagnose visuospatial neglect and impairments in responding to sensory stimuli in the environment.^20,83,84^ To begin, visuospatial neglect is a highly heterogenous disorder which can present in different forms such as spatial and non-spatial.^30,35,85^ The design of clinical tools like the BIT was thus aimed at providing a broad assessment of visuospatial neglect.^42,86^ We could have compared RT impairment and the spatial components of the BIT (cancellation tests) to provide a more accurate comparison of RT impairment and this clinical tool, but as the spatial components of the BIT encompass the majority of the BIT scoring system (130 out of 146), our results would likely not change. Another key difference is that the BIT does not consider the time required to respond to sensory stimuli permitting as much time as possible to survey the sheet of paper. In contrast, the key point of RT tasks is to capture the ability to quickly orient to stimuli. This is clearly demonstrated by Manly *et al.*^84^ They recorded video of patients completing the Star Cancellation test to examine temporal performance such as cancellation pace and time on task. Importantly, they found that the number of targets cancelled did not correlate with any of their temporal measures. Hence, assessments that quantify RT impairment may be more sensitive than common clinical tests in identifying visuospatial neglect.^27,29,30,87,88^ Further, an intriguing feature of measuring RT impairment is that individuals with stroke can show large variation in their movement initiation with some trials performing within healthy control range. This has not been explicitly shown in our study but can be seen in the hand speeds of our exemplar with stroke. Longer or more variable RT, which cannot be captured in common clinical tests, may be important to identify as it may increase the risk of falls.^89,90^

Another important question is how RT impairment relates to ADL. As RT general was created to determine RT in all spatial directions, one could expect a relationship with ADLs as both are required for successfully interacting with the environment. In line with previous studies comparing ADLs and RT,^91,92^ our data identified that FIM and RT general were significant but weakly correlated. Further, comparing the motor subscale of the FIM did not identify stronger correlations (data not shown). Our interpretation of the low correlation is first due to the lack of any time restrictions in the FIM. Second, the scope of the FIM is meant to cover a broad range of functions such as brushing teeth, toileting and bladder control.

### Neural basis of reaction time impairments

Visuospatial neglect has been associated with damage to many cortical areas^61,93^ including the insula,^94‐96^ posterior parietal cortex (PPC),^97‐99^ STG and MTG.^94‐96,100^ As well, it has been suggested that damage to white matter tracts, such as the superior longitudinal fasciculus (SLF), IFOF and ILF, may be associated with visuospatial neglect.^101‐103^ We build on previous findings by identifying many of the same regions associated with RT impairment. Regions associated with RT asymmetry impairment were commonly related to visual pathways to visual cortex (optic radiation) and pathways from the occipital cortex to other cerebral lobes (IFOF and ILF). One region of particular interest is the insula and its role in body awareness.^104^ This region has been shown to have vast connections with many sensory regions^105,106^ and has been associated with neglect.^107,108^ RT general impairment was associated with lesions in the STG and MTG and provided further evidence for the role of these regions in general visuospatial attention.^33‐35,100,109,110^

Surprisingly, we did not identify the PPC or SLF as being associated with RT impairment despite the importance of these regions in generating reaches^111‐114^ and visuospatial neglect.^97,99,115^ This may be partially explained by Karnath *et al.*^116^ suggesting lesions of the temporal rather than the parietal cortex impair performance in tasks requiring spatial awareness. Our results also highlight this association between lesions of the temporal lobe and RT impairments.

Our analysis identified that individuals with visual field deficits demonstrated impairment in both RT asymmetry and general. One would expect individuals with visual field deficits to display impairment solely in RT asymmetry as lesions to primary visual areas or visual pathways to these regions can impair visual input for specific hemifields.^117‐119^ Our data highlight a substantial proportion of these individuals presented with RT general impairment suggesting the presence of visual field deficits does not exclude potential RT impairment. This point can be further highlighted in a study conducted by Gaffan and Hornak^120^ in non-human primates. In this study, they performed optic tract lesions and forebrain commissurotomy to examine which lesions could produce neglect-like symptoms. They found that combined lesions produced substantial lateralized visuospatial neglect rather than lesions of the optic tract or forebrain commissurotomy alone.

### Sex, reaction time and stroke

It has been suggested that sex can have an impact on visuospatial ability. Studies have identified differences in RT for males and females with studies demonstrating faster RT in males than females ^48,121^ but others identify only minor differences.^122^ In our study, we attempted to reduce the effects of sex, handedness and movement direction by generating normative models based on healthy control data. Our models identified slower RT for targets towards 45° in males compared to females but similar RT in all other directions. An analysis of RT across all directions identified marginally faster RT for males.

An interesting question is whether the influence of stroke on visuospatial ability, shown to differ for males and females, would also display a sex effect for RT impairment. To our knowledge, only a limited number of studies examined this. Godefroy *et al.*^123^ found no sex differences for simple and choice RT for individuals with stroke. Our analysis of RT for individuals with stroke found greater RT impairment in males than females but this was not significant.

### Limitations

Individuals with visual field deficits were identified through the confrontation technique, and thus the reported number may be an underestimate. We did not separate individuals with visual field deficits from our cohort for a couple of reasons. First, all these individuals failed the BIT. This is interesting to note as the BIT was designed to diagnose attentional impairment rather than impairments in visual input. Second, as mentioned previously, these individuals were impaired in both RT asymmetry and RT general. Of note, 6 of 14 individuals with RHD and visual field deficits had RT general impairment, 3 of which did not display RT asymmetry impairment. Regardless, future studies should complete a more thorough examination to better understand the relationship between visual field deficits and RT impairments.

Our imaging identified many locations implicated in RT impairment after RHD than LHD. As mentioned previously, it is thought that there is a dominance for the right hemisphere in visuospatial control for the left and right sides of space whereas the left hemisphere only controls the right. Therefore, RT impairment due to left hemisphere lesions would be less prominent than right hemisphere lesions. Further analysis separating individuals with RHD and LHD did not find results that were substantially different and did not alter the message of the paper (data not shown). Additionally, while our sample was relatively large, there were fewer individuals with left hemisphere lesions (*n* = 103) as compared to right hemisphere lesions (*n* = 162).

## Supplementary Material

fcad066_Supplementary_Data

## Data Availability

The Kinarm data that support the findings of this study are available on request from the corresponding author. Clinical data are not publicly available due to their containing information that could compromise the privacy of research participants.

## Abbreviations

AAL =: automated anatomical labelling
ADC =: apparent diffusion coefficient
BIT =: Behavioral Inattention Test
CMSA =: Chedoke-McMaster Stroke Assessment Impairment Inventory
DWI =: diffusion-weighted imaging
FLAIR =: fluid-attenuated inversion recovery
IFOF =: inferior frontal–occipital fasciculus
ILF =: inferior longitudinal fasciculus
LHD =: left hemisphere damage
MoCA =: Montreal Cognitive Assessment
MTG =: middle temporal gyrus
MNI =: Montreal Neurological Institute
PPC =: posterior parietal cortex
RHD =: right hemisphere damage
RT =: reaction time
SLF =: superior longitudinal fasciculus
sROI =: statistical region of interest
STG =: superior temporal gyrus
VGR =: visually guided reaching
