# Supplementary material for: Directional and general impairments in initiating motor responses after stroke
Source: Brain Commun. 2023 Mar 20;5(2):fcad066. doi: 10.1093/braincomms/fcad066 (PMC10087022; doi:10.1093/braincomms/fcad066)
Supplement: fcad066_Supplementary_Data [file fcad066_Supplementary_Data.pdf]

Supplementary Material

Subtraction Analysis Method

A subtraction analysis was performed in MRIcron[124] to evaluate voxel-wise differences in lesion frequencies between participants who were deemed impaired and normal on each behavioural parameter. Briefly, at every voxel, the proportion of participants with lesions in that voxel, who passed the parameter, were subtracted from the proportion of participants with lesions in that voxel, who failed the parameter. Thus, leaving lesioned voxels where the relative proportion of participants who failed the parameter was greater than that which passed the parameter.

Subtraction Analysis Results

Supplementary Figure 2A highlights the lesion overlap across all participants with stroke. The subtraction analysis for both Left and Right Arm RT Asymmetry (Supplementary Figure 2B and Supplementary Figure 2C) implicated the insula, with the latter also being associated with lesions to the right Heschl's Gyrus and rolandic operculum. For the Left Arm RT General, the subtraction analysis highlighted lesions spreading around the right insula as more common regions in those impaired (Supplementary Figure 2D). For the right arm, a cluster of lesions in the right temporal lobe were associated with impairment (Supplementary Figure 2E). These regions included the right STG and MTG and temporal pole.

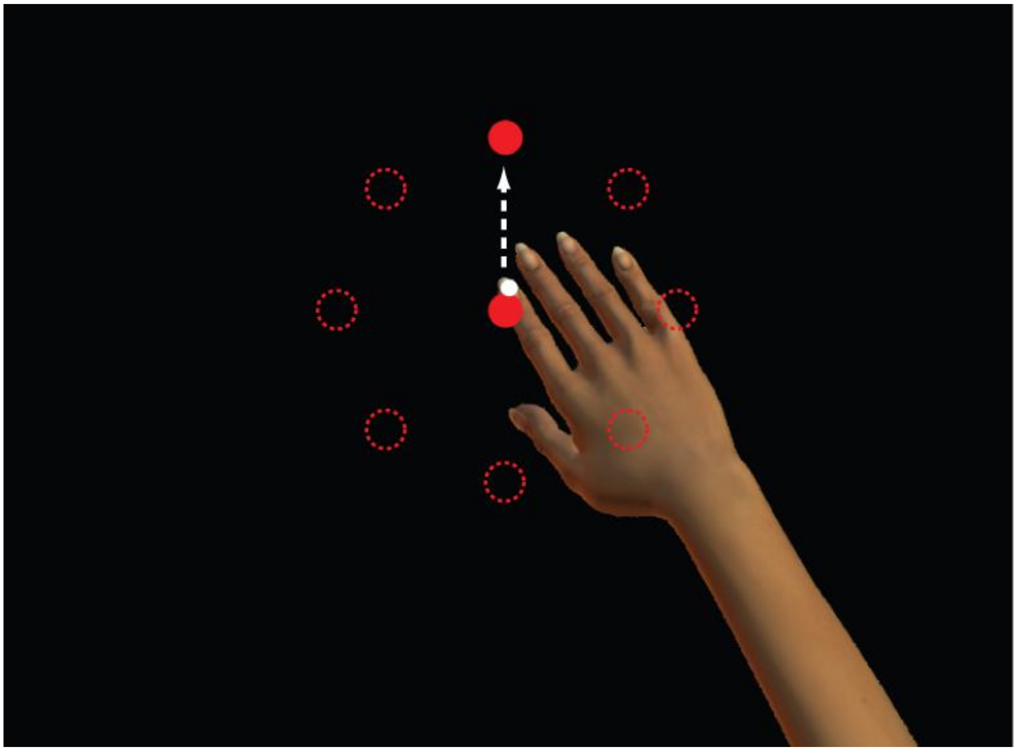

Supplementary Figure 1: Participant performing the Visually Guided Reaching task. This centre-out reaching task was performed in the horizontal plane where visual feedback of the finger (white dot), central start target (lower filled red dot), and peripheral target (upper filled red dot) are displayed. Direct vision of the limb was occluded with a physical barrier. The unfilled red dashed circles indicate peripheral target locations that were presented in other trials. The white dashed arrow indicates the direction of hand motion from the centre to the peripheral target and was not presented during the task.

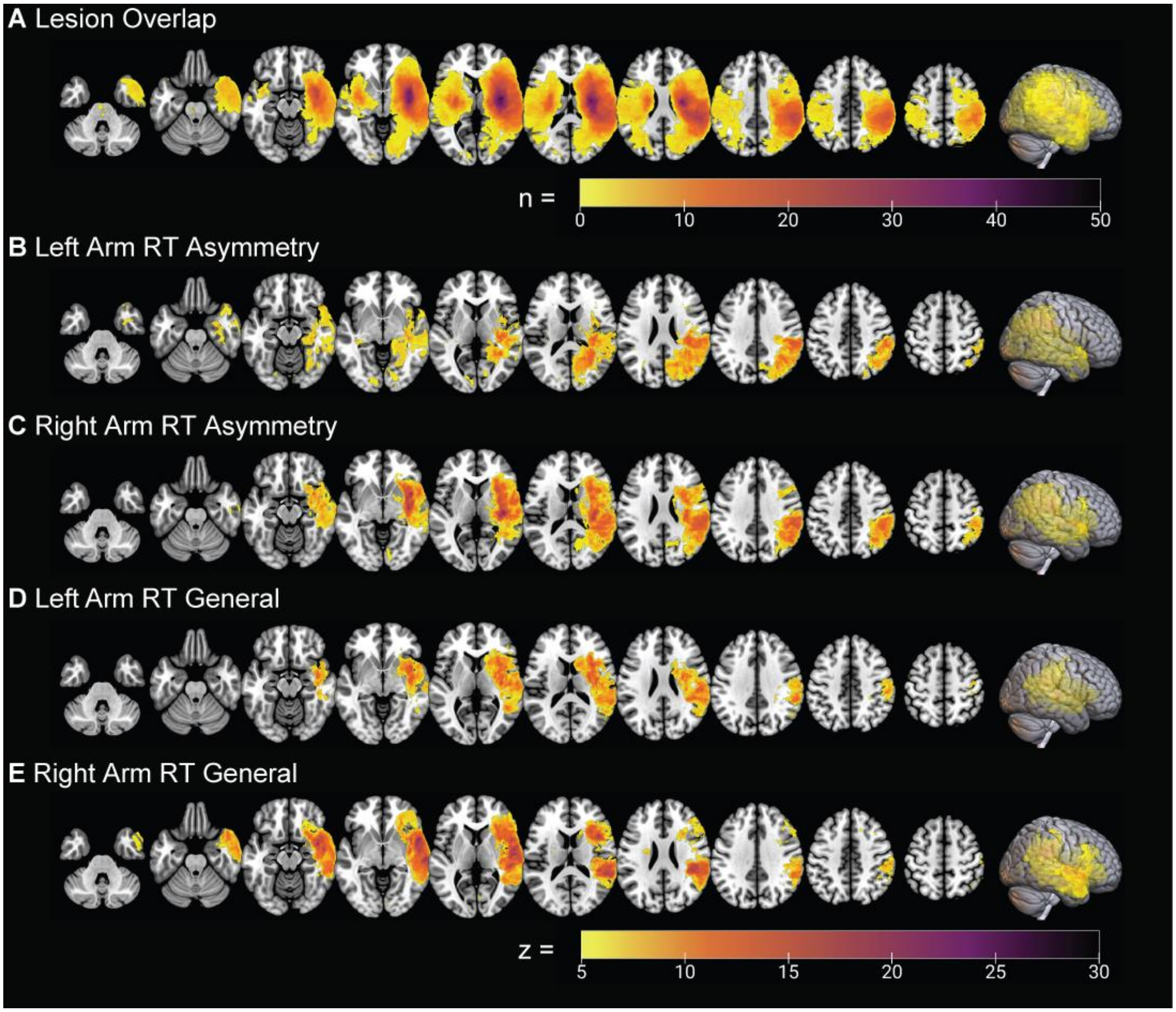

Supplementary Figure 2: Lesion overlap maps of all stroke individuals. (A) Overlap of lesion location for all individuals with stroke (*n* = 201). **(B) and (C)** Subtraction analysis lesion location for those impaired in RT Asymmetry of the left and right arms, respectively. Note, RT impairments were defined based on transformed data. **(D) and (E)** sROI maps for those impaired in RT General in the left and right arms, respectively.
